# Alkoxysilane-Mediated Decoration of Si Nanowires Vertical Arrays with Au Nanoparticles as Improved SERS-Active Platforms

**DOI:** 10.3390/ijms242316685

**Published:** 2023-11-24

**Authors:** Maria Josè Lo Faro, Ileana Ielo, Dario Morganti, Antonio Alessio Leonardi, Sabrina Conoci, Barbara Fazio, Giovanna De Luca, Alessia Irrera

**Affiliations:** 1 Dipartimento di Fisica e Astronomia “Ettore Majorana”, Università degli Studi di Catania, 95123 Catania, Italy; mariajose.lofaro@unict.it; 2 Istituto per la Microelettronica e Microsistemi, CNR-IMM Catania Università, 95121 Catania, Italy; 3 Dipartimento di Scienze Chimiche, Biologiche, Farmaceutiche, ed Ambientali, Università degli Studi di Messina, 98166 Messina, Italy; ileana.ielo1@unime.it (I.I.); dario.morganti@unime.it (D.M.); sabrina.conoci@unime.it (S.C.); 4 URT LAB SENS CNR and Beyond Nano, CNR, 98166 Messina, Italy; antonioalessio.leonardi@cnr.it (A.A.L.); barbara.fazio@cnr.it (B.F.); 5 Istituto per la Microelettronica e Microsistemi, CNR-IMM Zona Industriale, 95121 Catania, Italy

**Keywords:** Silicon nanowires, Au nanoparticles, SERS, surface functionalization, 3D template

## Abstract

The search for improved transducers to fabricate better-performing (bio)sensors is a challenging but rewarding endeavor aiming to better diagnose and treat diseases. In this paper, we report on the decoration of a dense vertical array of ultrathin silicon nanowires (Si NWs), produced by metal-assisted chemical etching, with 20 nm gold nanoparticles (Au NPs) for surface-enhanced Raman scattering (SERS) applications. To optimize the production of a uniform 3D SERS active platform, we tested different Si NW surface functionalizations with various alkoxysilanes before Au decoration. Scanning electron microscopy investigations confirm that Au NPs decorate both bare and (3-glycidiloxypropyl)trimethoxysilane (GPTMS)-modified Si NWs with a high surface coverage uniformity. The SERS response of the decorated NWs was probed using a model dye system (methylene blue; MB) at 633 and 785 nm excitation wavelengths. The GPTMS-modified NWs present the highest enhancements of 2.9 and 2.6 for the 450 cm^−1^ and 1625 cm^−1^ peaks under 785 nm excitation and of 10.8 and 5.3 for the 450 cm^−1^ and 1625 cm^−1^ peaks under 633 nm excitation. These results demonstrate the perspective role of Si NWs decorated with Au NPs as a low-cost 3D SERS platform.

## 1. Introduction

Optical biosensors can rely on several types of signals for the detection of the bioanalyte binding event: light absorption, emission, or scattering and surface plasmon resonance, as well as surface-enhanced raman scattering (SERS), to name a few [1].

In this framework, surface nanostructuring by plasmonic nanoparticles (NPs) ensembles is a well-established method for fabricating surface-enhanced raman scattering (SERS) active substrates [2,3,4,5]. Noble metals such as gold and silver have always been constituents of choice for plasmonic nanostructures, given the fine tunability of their morphological and optical features [6,7,8,9,10,11]. A widespread wet-chemical method for synthesizing noble metal NPs involves forming colloidal solutions, possibly employing different precursors, reductants, solvents, and NP capping agents [12,13]. SERS-active transducers can then be fabricated by depositing the plasmonic nanostructures onto the substrates of interest via physical or chemical methods, with examples of excellent control in the geometry, coverage, and roughness (among other parameters) of the active surfaces [14,15,16].

Compared to flat SERS-active substrates, the decoration of 3D architectures like nanowire arrays is of particular importance in enhancing and controlling the metal NP SERS response [17,18]. Three-dimensional nanostructures provide an improved surface-to-volume ratio, offering the advantage of hosting a higher number of plasmonic nanoparticles compared to flat surfaces, hence improving the SERS enhancement [19,20,21,22]. As well documented in the literature, three-dimensional (3D) assemblies possess enhanced performances of plasmon-based sensing and imaging applications due to the creation of highly localized electromagnetic fields (i.e., hot-spots) fostering ultrasensitive SERS detection of biomolecules at lower concentrations compared to flat surfaces from one to three orders of magnitude [20,23].

The study of SERS substrates based on silicon is of particular notice due to its availability, low cost, nontoxicity, and well-developed industrial technology [24]. Indeed, silicon nanowires have been extensively explored for sensing applications due to their unique advantages in terms of biocompatibility, convenient surface modification with various functional groups, high surface-to-volume ratios, fast response, and good reproducibility [25,26]. As reported in the literature, the wide range of synthetic methods makes Si NWs easy to fabricate, and their physical and chemical features promote the observation of incredible geometrical, optical, and electrical performances [27,28,29]. In the SERS scenario, metal-modified Si NWs are strongly attractive compared to traditional flat SERS architectures for their rapid detection of low concentrations of molecules. As a consequence, many groups have taken advantage of Si NWs decorated with metal NPs for the SERS detection of pesticides [30], bacteria like *E. coli* [31,32], or biomolecules [33,34], as well as for DNA detection down to 1 pM [35].

The 3D structure of Si NWs allows them to harbor a higher number of metal NPs than flat substrates, resulting in a very elevated hot-spot density of optically coupled NPs, strongly amplifying the Raman signal of the analytes. The modulation of the radii, interparticle distance, and the density of the NPs provides the necessary flexibility to control the NP plasmon resonances with a particular interest in (bio)sensing [34,36,37].

Many efforts over the years have been devoted to the large area decoration of Si NW vertical arrays, preferably with silver and gold NPs, not only for their SERS response but also due to their excellent integration with Si-based systems [17], chemical routes being often the preferred approaches to this task [38]. For example, the NP growth can be induced onto the Si surface via redox reactions in the presence of AgNO_3_ or NaAuCl_4_ solutions, where electrons are transferred from the zero-valent Si surface to the Ag^+^ and Au^3+^ ions, inducing the oxidation of Si to Si^4+^ [38]. Physical routes are also employed in the literature for the metal decoration of Si NWs [39,40]. For example, Li et al. obtained the formation of crystalline Au NPs with a 5 nm radius onto thermally grown Si NWs with 20−50 nm in diameter and more than 10 μm in length through sputtering at room temperature and pressure of 1 × 10^−2^ Torr [40]. However, the finer control that characterizes the physical techniques often comes at higher instrumentation costs. Ultimately, chemical routes are cheaper and faster than the physical syntheses, although at the cost of a less controlled NP distribution. A uniform and dense NP deposition is easily obtained only when single NWs have been previously dispersed, while an array decoration is complicated, e.g., it often results in the deposition of clusters at the NW tips [41,42]. Besides, compared to other physical approaches like laser ablation, sputtering, or evaporation, the decoration of Si NWs with Au NPs upon their chemical functionalization allows the preservation of their characteristic fractal features.

In this paper, we exploited the role of surface modifications of a dense and vertical array of ultrathin Si NWs with various alkoxysilanes before Au decoration. Alkoxysilanes are commonly employed to impart new functions to silicon-based surfaces, whether they be particles or macroscopic systems [43,44]. By carefully choosing the functional groups to be exposed, we are able to boost the coverage of the Si NW surface by plasmonic nanoparticles and amplify further the SERS signals of a (bio)analyte of interest, thus fabricating improved SERS transducers for biosensing applications.

## 2. Results and Discussion

### 2.1. Silicon Nanowires Fabrication

As reported in the literature [27,45], a vertical array of Si NWs is fabricated by metal-assisted chemical etching. This approach allows us to obtain an ultra-thin Si NW array with a high density of 10^12^ NW/cm^2^, a length of 3.5 ± 0.2 µm, and average NW diameter of 7 ± 1 nm. Moreover, this fabrication method allows us to tune the 2D fractal arrangement of the NWs across the plane by varying the Si substrate (100 or 111) type or the Au film thickness [27]. Indeed, the self-similarity and scale invariance of fractals are extended over a broad length scale range (from 100 nm up to a few microns) fostering extended optical trapping across a broad wavelength scale, from 250 up to 1100 nm [46]. To this end, a UV/ozone-cleaned Si wafer is etched in an aqueous HF solution to remove the native oxide, and a 2 nm gold coating is deposited on the Si wafer via electron beam evaporation under high vacuum conditions. The above-described Si NW array is then produced using the percolative thin Au film as a template catalyst during the subsequent metal-assisted chemical etching. This process occurs in an aqueous solution of H_2_O_2_ and HF at the Au/Si interface due to the materials’ electronegativity difference. In particular, Si oxidation via H_2_O_2_ is followed by HF removing SiO_2_, the residual Au precursor being removed by a gold etchant solution. After their production, a 2 nm native oxide layer develops on the Si NW surface [45]. In order to handle substrates with comparable Si NW surface coverage, 0.5 × 0.5 cm^2^ samples are selected from the same production batch to later be decorated with Au NPs. To this aim, both bare and surface-modified Si NW substrates are employed.

### 2.2. Alkoxysilanes Grafting of the Si NW Surface

**Figure 1:**
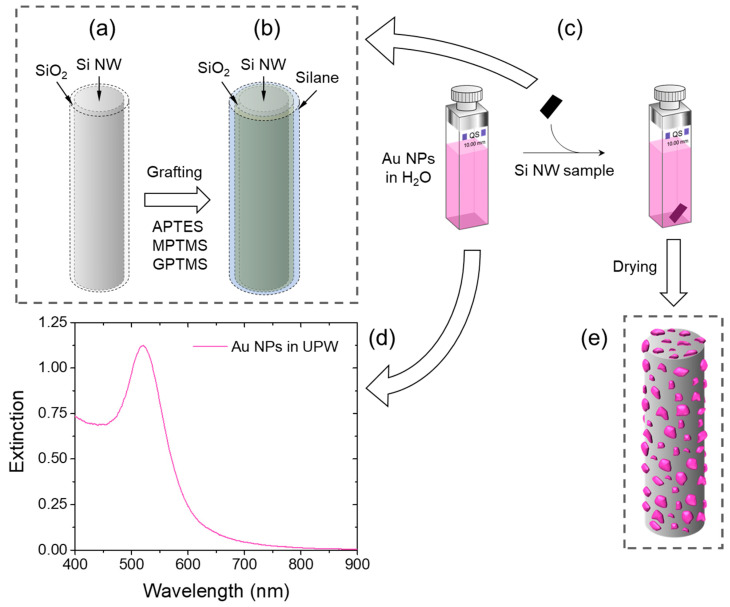
Scheme depicting the decoration with Au NPs (**a**) before and (**b**) after the surface modification of Si NWs with APTES, MPTMS, and GPTMS. (**c**) Nanoparticle infiltration into the Si NW array. (**d**) Extinction spectrum of a waterborne Au colloid (10^−3^ M in Au(III) ions). (**e**) Final decoration of Si NW with Au NPs.

**Figure 2:**
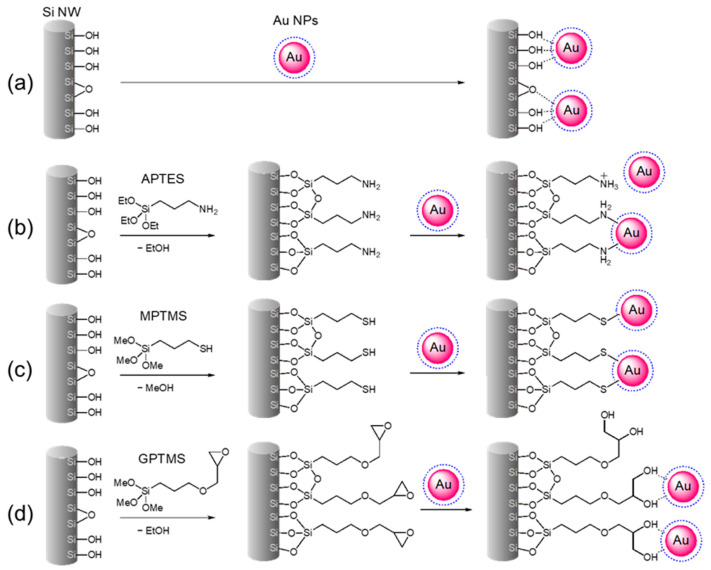
Schematic representation of the decoration with Au NPs of Si NWs, before (**a**) and after their surface modification with three functional groups APTES (**b**), MPTMS (**c**), and GPTMS (**d**).

The native oxide layer covering Si NWs is subjected to grafting with three different trialkoxysilanes (Figure 1a,b and Figure 2): (3-aminopropyl)triethoxysilane (APTES-Si NWs), (3-mercaptopropyl)trimethoxysilane (MPTMS-Si NWs), and (3-glycidyloxypropyl)trimethoxysilane (GPTMS-Si NWs). These surface functionalizations are easily obtained by immersing the Si NW substrates into toluene solutions of the three alkoxysilanes (5% *v/v*, 10 mL) for 24 h under gentle shaking at 25 °C. After thorough washing with neat toluene, ethanol, and ultrapure water (UPW; thrice in each solvent), the samples are extracted from the latter and dried in air immediately before placing them in the Au NP colloidal solution (Figure 1c and Figure 2). This surface functionalization technique is very well-established, and it provides Si surfaces, either pristine or covered by SiO_2_, with an extremely rich chemistry thanks to the numerous organosilane derivatives [47].

### 2.3. Decorating Si NWs with Gold Nanoparticles

Au sol was obtained via the Turkevich method, i.e., by reducing Au^3+^ ions with citrate in boiling water [48,49]. The resulting colloidal solution presented the typical magenta color, characterized by a plasmon absorption band at about 520 nm (Figure 1d; starting Au(III) ion concentration: 1 mM) and a pH of 8.2. The Au NP size distribution was determined via dynamic light scattering to be monomodal, with a mean hydrodynamic diameter of 22 ± 4 nm.

The decoration with Au NPs was then attained by immersing the bare and surface-modified Si NW substrates in the Au nanocolloid for 4 h under gentle shaking at 25 °C (Figure 1c−e), followed by thorough washing in neutral UPW. The decoration of bare substrates is guided mainly via physisorption, whereas with functionalized Si NW, the process is designed to exploit typical supramolecular interactions, leading to self-assembled monolayers (SAMs) on gold.

Thiols and amines are well-known capping agents for noble metal nanostructured surfaces, explaining the choice of APTES and MPTMS as surface modifiers for decorating Si NWs with Au NPs. As indicated by results in the literature, alkylamines interact with Au primarily through -NH_2_ moieties and, to a lesser account, through protonated -NH_3_^+^ groups (Figure 2b) [50], whereas gold–sulfur interactions can be represented as homolytic Au(0)-thiyl bonding with the formation of H_2_ (Figure 2c) [51].

In its turn, the epoxide ring of GPTMS readily undergoes a ring-opening reaction, forming a vicinal diol molecular pattern. Molecules with 1,2-/1,3-diol patterns, e.g., catecholates or polyvinylalcohol molecules, are known to chelate the Au surface and stabilize NP colloids (Figure 2d) [52,53]. Moreover, citrate-stabilized Au NPs may also bind the 3-glycidyloxypropyl moiety through covalent interactions if the same citrate molecules of the capping layer causes the epoxide ring-opening reaction [54].

### 2.4. Electron Microscopy Analysis of Au NP-Decorated Si NWs

**Figure 3:**
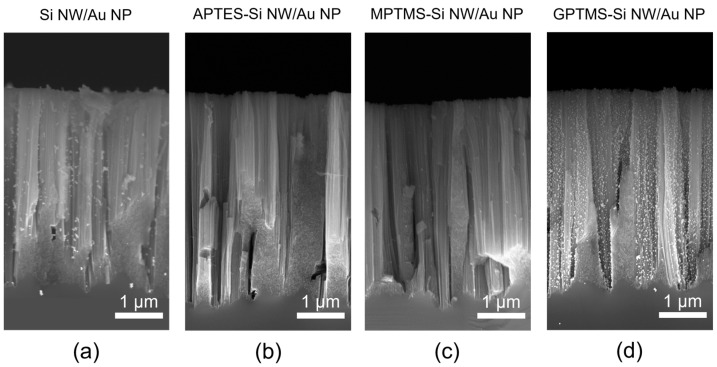
Cross-section scanning electron microscopies of bare (**a**) and surface-modified (**b**–**d**) Si NWs decorated with Au NPs. Surface modifications: APTES (**b**), MPTMS (**c**), and GPTMS (**d**).

All the NP-decorated samples are then investigated by scanning electron microscopy (SEM) in cross-section, as displayed in Figure 3. The micrographs’ comparison shows that bare Si NWs present an inhomogeneous coverage with Au NPs being locally isolated or coalesced (Figure 3a), while none or only a few particles are visible on APTES-Si NW and MPTMS-Si NW (Figure 3b and Figure 3c, respectively). In contrast, NW samples modified with GPTMS present a uniform and homogeneous NP distribution on their vertical wall (Figure 3d). This evidence can be tentatively explained considering that, under the employed experimental conditions, the interactions between the water molecules and the citrate layer stabilizing the Au NPs are pretty strong, making it harder for the –SH and –NH_2_ moieties to reach and bind covalently to the Au surface.

**Figure 4:**
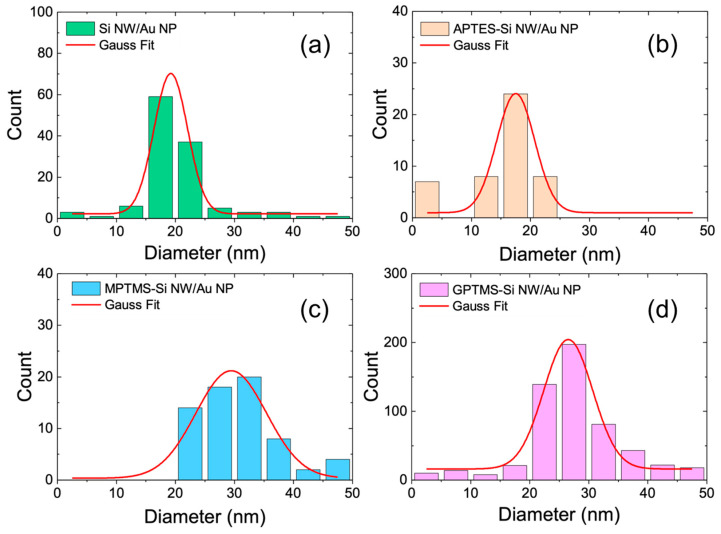
The statistical analysis of the Au NP radii is reported for bare (**a**) and surface-modified (**b**–**d**) Si NWs decorated with Au NPs. Surface modifications: APTES (**b**), MPTMS (**c**), and GPTMS (**d**). The data are fitted with Gaussian profiles as red lines.

The size distributions of the Au NPs decorating the four Si NW samples were obtained through the analysis of the SEM micrographs and are reported in Figure 4 as bare Si NWs/Au NPs (green), APTES-Si NWs/Au NPs (orange), MPTMS-Si NWs/Au NPs (light blue), and GPTMS-Si NWs/Au NPs (magenta). Each histogram is fitted with a Gaussian distribution (red lines), and the average values for the Au NP diameters are reported in Table 1 for all samples, their differences are statistically irrelevant, and they agree well with the average size of (22 ± 4) nm measured in solution via DLS.

### 2.5. Surface Enhanced Raman Scattering Analysis

**Figure 5:**
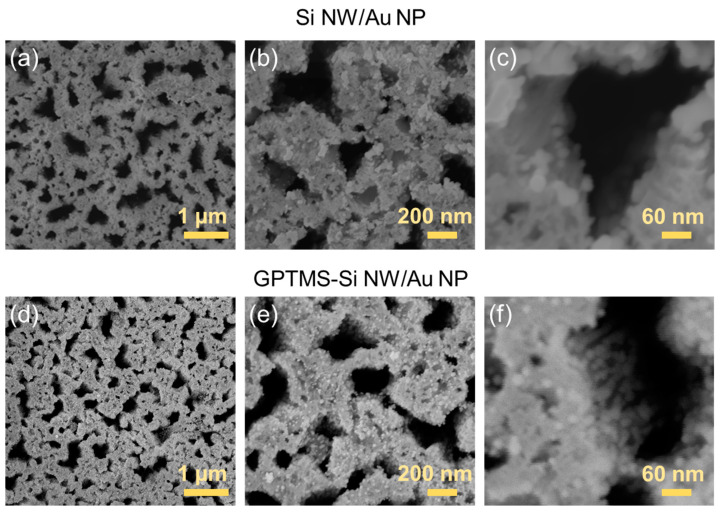
Plan-view scanning electron microscopies of Si NWs decorated with Au NPs before (**a**–**c**) and after (**d**–**f**) their surface modifications with GPTMS at (**a,d**) low, (**b,e**) medium, and (**c,f**) high magnification conditions.

At a preliminary screening, the enhancement of the Raman scattering via APTES- and MPTMS-Si NWs/Au NPs is not noticeable, probably given the low amount of Au NPs decorating the Si NWs. Hence, any further investigation is only focused on bare Si NWs/Au NPs and GPTMS-Si NWs/Au NPs nanoarchitectures. Figure 5 shows more detailed plan-view SEM micrographs of the bare Si NWs/Au NPs (a−c) and GPTMS-Si NWs/Au NPs (d−f) reported at increasing magnifications. The comparison between the two samples, mainly at medium (Figure 5b,e) and high (Figure 5c,f) magnifications, clearly shows the difference in the NW surface coverage across the plane, pointing to the advantages offered by GPTMS grafting, which provides a better binding of the citrate-stabilized Au NPs onto the Si NW walls.

**Figure 6:**
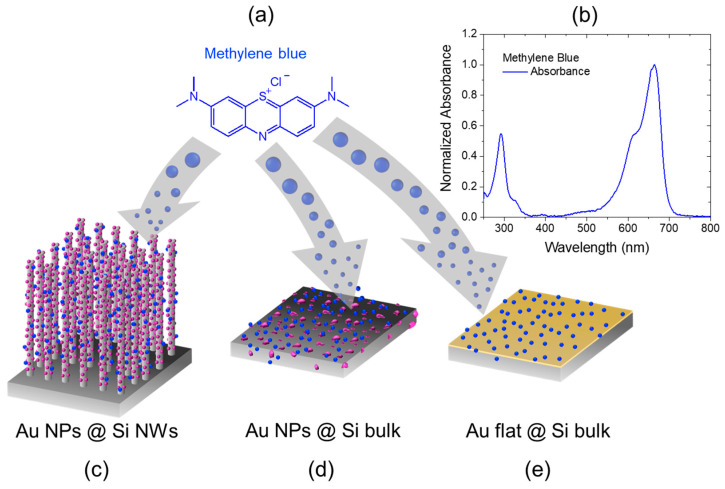
Sketch depicting the preparation of the SERS samples through the absorption of MB (**a**), whose normalized UV/vis spectrum is reported in (**b**), on bare and GPTMS-functionalized Si NWs/Au NPs (**c**), bare and GPTMS-functionalized Si bulk-covered with Au NPs (**d**), and a flat Au film onto Si bulk deposited via sputtering (**e**).

The SERS properties of Si NW/Au NP and GPTMS-Si NW/Au NP samples are tested using methylene blue (MB, Figure 6a), a probe molecule often used to investigate the efficiency/enhancement of several types of SERS substrates [55]. This dye is known to spontaneously absorb onto various surfaces to such a great extent that it can even be used to measure the surface area of powdered or porous samples [56,57,58]; its interaction with the surfaces is thus ensured without needing any further functionalization.

Figure 6 schematizes the preparation of a set of three types of samples: bare and GPTMS-functionalized Si NWs/Au NPs (c), bare and GPTMS-functionalized Si bulk-covered with Au NPs (d), and thin flat film of Au deposited via sputtering and used as a reference (e). All samples were immersed in a solution of MB (10^−2^ M) for 150 min, then thoroughly washed in UPW and dried overnight. The top panel in Figure 6b reports the normalized absorbance spectra of MB, showing a major absorption peak at about 650 nm accompanied by a shoulder at 612 nm. The origin of both features is quite debated in the literature [59,60]. Recently, Kostjukova et al. attributed the first component to a π → π* electronic transition and the shoulder to higher vibronic transitions [60].

**Figure 7:**
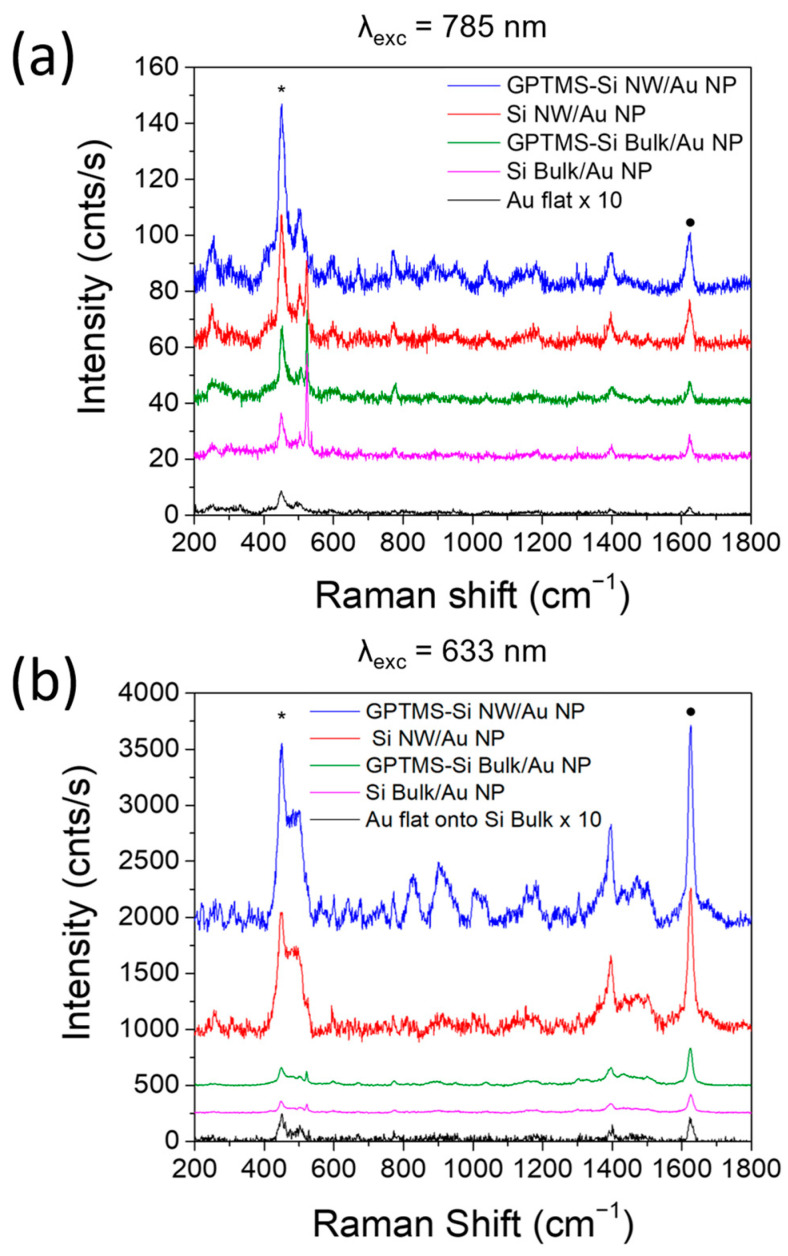
SERS spectra of MB acquired on bare (red line) and GPTMS-functionalized (blue line) Si NWs/Au NPs, bare (magenta line) and GPTMS-functionalized (green line) Si bulk-covered with Au NPs, and Au flat film (black line) under excitation wavelengths of 785 nm (**a**) and 633 nm (**b**). The 450 and 1625 cm^−1^ bands are marked with a black star and dot, respectively.

The SERS analysis was carried out under excitation wavelengths of 785 nm and 633 nm, as reported in Figure 7a and Figure 7b, respectively. As with other dyes, MB presents fluorescence emission from singlet states centered at about 686 nm in the 650–900 nm range, and it is generally susceptible to modification due to external conditions, such as pH, solvent type, or self-aggregation, leading to quenching [59]. The fluorescence background was removed from SERS spectra for clarity using a second-order polynomial fitting. MB SERS spectrum features several vibrational bands in the 400–1650 cm^−1^ region, the most intense being found at 450 and 1625 cm^−1^ and marked with a black star and dot, respectively. The first band is more intense under 785 nm excitation and is associated with the C–N–C stretching of the two amino groups. The latter band at 1625 cm^−1^ becomes dominant under excitation at 633 nm and is assigned to C–C ring stretching and C–N ring stretching. Figure 7 presents the SERS spectra of MB acquired on bare (red line) and GPTMS-functionalized (blue line) Si NWs/Au NPs, bare (magenta line) and GPTMS-functionalized (green line) Si bulk-covered with Au NPs, and Au flat film (black line) under excitation wavelengths of 785 nm (a) and 633 nm (b). For both excitations, the SERS intensities of the two MB main vibrational bands (at about 450 and 1625 cm^−1^) are reported in Table 1 for all the samples. SERS enhancement factor (EF) is defined as the ratio of SERS and Raman intensity normalized using the ratio of the Raman and SERS concentrations (I_SERS_/I_Raman_ × C_Raman_/C_Sers_) while taking into account the laser power density at the focal point and the integration time. A reference for Raman measurements was established using a standard flat Au film produced through sputtering. Under the hypothesis that MB adsorbs on Au with the same affinity regardless of whether the metal surface was flat or nanostructured, the dye concentration used in the SERS experiment on decorated Si NWs or the flat Au film was considered comparable (C_Raman_ = C_Sers_). We thus calculated the EF for each sample at two different excitation wavelengths (633 nm and 785 nm) and for the two MB characteristic bands (450 cm^−1^ and 1625 cm^−1^). This comparison confirms that the GPTMS surface functionalization of both Si bulk and Si NWs allows a stronger enhancement of the Raman scattering with respect to the corresponding untreated surfaces, as it provides a more efficient and homogeneous decoration of the surfaces with Au NPs.

The contribution to the SERS effect due to the 3D nature of the Si NW substrate (Table 2, EF columns) is made evident when comparing the normalized ratio of the SERS intensity for the GPTMS-Si NW/Au NP vs. the GPTMS-Si Bulk/Au NP. Under 785 nm excitation, the 3D nanoarchitecture presents an EF of 2.9 for the band at 450 cm^−1^ and of 2.6 for the signal at 1625 cm^−1^, whereas values of 10.8 and 5.3 are obtained for the same two signals, respectively, when excited at 633 nm. A similar improvement is highlighted by comparing bare Si NWs/Au NPs to Si Bulk/Au NPs, with enhancement factors of 2.5 at 450 cm^−1^ and 2.4 at 1625 cm^−1^ under 785 nm excitation and 10.4 at 450 cm^−1^ and 7.4 at 1625 cm^−1^ under 633 nm excitation. This effect may easily be connected to the much larger surface area made available for Au NP decoration in the presence of the three-dimensional nanostructured surface provided by the Si NWs compared to that of a flat surface.

It is well known that capped nanoparticles generally exhibit lower enhancement factors, ranging from 1 to 3 orders of magnitude maximum, compared to uncapped nanoparticles, as SERS is a near-field effect. When surveying the literature, Vega et al. showed an EF of 10^3^ using citrate-protected Au NPs with a size of (21.9 ± 2.3) nm for the detection of Rhodamine 6G [61]. A similar enhancement has been shown by Darienzo et al., who report an EF of 10^3^ for malachite green dye using spherical citrate-capped Au NPs with a size of 60 nm [62]. Bartschmid et al. reported the SERS performances of Si NW arrays decorated with Au NPs obtained by Turkevich citrate-route with EF of about 12; two times higher than the one attested with the same NPs on flat Si functionalized with APTES and MPTMS to improve surface coverage [63]. In this framework, the EF of ca. 11 observed with our system points to the perspective role of our GPTMS-functionalized Si NWs decorated with citrate-capped Au NPs as 3D SERS platform.

## 3. Experiment Details

### 3.1. Materials

Hydrofluoric acid (HF, 50%), KI gold etchant solution, methylene blue (MB, powder), tetrachloroauric(III) acid trihydrate (HAuCl_4_; 99%), trisodium citrate dihydrate (Na_3_C_6_H_5_O_7_, 99–100%), (3-aminopropyl)triethoxysilane (APTES; 99%), (3-mercaptopropyl)trimethoxysilane (MPTMS; 95%), and (3-glycidyloxypropyl)trimethoxysilane (GPTMS; 98%) were purchased at Sigma Aldrich (Milan, IT). Ultrapure water (UPW) was purchased at VWR (Milan, IT). Si wafers were purchased from Siegert Wafer (Aachen, DE). All chemicals were used without any further purification.

### 3.2. Au Nanoparticles Synthesis 

The synthesis was carried out according to literature protocols [49]. In particular, 40 mg of HAuCl_4_ was dissolved in 100 mL of UPW (solution 1). 0.5 g of Na_3_C_6_H_5_O_7_ was dissolved in 50 mL of UPW (solution 2). Solution 1 was heated to T = 100 °C for 12 min in a sand bath, then 2 mL of solution 2 was added dropwise to the boiling solution 1. A color change of the solution from pale yellow to ruby red was observed. The pH of the colloidal solution measured as 8.2.

### 3.3. Dynamic Light Scattering Measurements 

The mean nanoparticle size was determined via dynamic light scattering (DLS). Measurements were performed at 25 °C using a Malvern Panalytical mod. Zetasizer Nano ZS instrument equipped with a He-Ne laser (4 mW, λ_0_ = 632.8 nm). A 173° scattering angle was employed. Au NP colloids were analyzed as synthetized. The mean diameter values measured for the various Au NP batches synthetized during the present study were consistent within experimental error with the 22 ± 4 nm value reported in the Section 2.

### 3.4. Si nanowires Synthesis 

The 100-crystalline Si wafers were cleaned via a 3 min UV-ozone treatment and then dipped in a 5% HF aqueous solution. The vertical array of Si NWs was obtained via metal-assisted chemical etching using a 2 nm thick layer of gold deposited as a catalyst for the process via an electron beam evaporator from Kenosystec. The reproducibility of the Au film through electron beam evaporation is very high, as the machine is equipped with quartz balance able to resolve the deposition of 1 Å. Once the deposition conditions of ultra-high vacuum (10^−8^ mbar) are achieved, the current filament is set at the value of 50 mA to melt the Au 99.999% pellet and obtain a thickness of 2 nm with a uniformity tolerance of 5% across the surface of a 4′’ wafer. As is well known, metal-assisted chemical etching is a robust procedure based on the optimization of the two competing processes of oxidation and etching, mediated by the HF:H_2_O_2_ ratio concentration in the aqueous solution. Once the solution is calibrated, setting the etching time provides the desired length. In our case, the Au-deposited samples were etched in an aqueous solution of HF:H_2_O_2_ (5:0.44 M) for 20 min to obtain a formation of 3.5 ± 0.2 µm long Si NWs. These were then rinsed in a KI-based gold etchant solution to obtain a metal-free surface, followed by three further 2 min rinsing in water [29,45].

### 3.5. Si Nanowires Surface Functionalization 

The pristine Si NWs were then surface-modified via treatment with APTES, MPTMS, and GPTMS to form a coupling layer between the oxidized surface of the Si NWs and the Au NPs. Before reacting with the three alkoxysilanes, the Si NW samples were subjected to a cleaning protocol to guarantee the removal of any contamination and promote the uniform formation of an external silicon oxide layer. Specifically, the samples were first cleaned in an isopropanol bath (2 min), rinsed in water (2 min), exposed to UV ozone treatment (5 min), washed in water (2 min), and finally dried via nitrogen flow at RT. Then, the cleaned Si NWs were surface-modified using a wet-chemical treatment with APTES, MPTMS, or GPTMS toluene solutions (5%, *v/v*, 10 mL). After surface grafting, the samples were washed in neat toluene, ethanol, and UPW (trice in each solvent). The silane-treated samples were immersed in 2 mL aliquots of the Au NP colloids at 25 °C under gentle shaking. The Au-decorated Si NW samples were washed thrice in UPW to remove all the unbound NPs and finally dried in air.

### 3.6. Scanning Electron Microscopy 

Morphological and structural characterizations of the NW samples were carried out using a Zeiss Supra 25 field-emission scanning electron microscope both in plan-view and cross-sections at high resolution with an InLens detector. The surface modification was confirmed through measurements for each sample, as displayed in Figure 4. The acquired electron microscope images were processed with Gatan DigitalMicrograph 3.6 software (https://www.gatan.com/products/tem-analysis/gatan-microscopy-suite-software, accessed on 23 November 2023), applying a threshold to properly select the Au nanoparticles, and their radii were measured via pixel counting per each sample, as reported in the statistical analyses of Figure 4.

### 3.7. Surface-Enhanced Raman Scattering Measurements

Raman and SERS measurements were performed using a He-Ne laser beam tuned at 633 nm and a solid-state laser at 785 nm. The laser beams were focused onto the samples through a 100× objective (NA = 0.9) with powers not exceeding 510 μW measured at the sample surface. The backscattered radiation was analyzed by means of an HR550 Micro-Raman Spectrometer (Horiba) equipped with a Peltier-cooled CCD (Sincerity) detector. Methylene Blue was selected as a probe molecule for SERS measurements. To study in detail the SERS response, four samples of 0.5 × 0.5 cm^2^ were soaked for 150 min in MB water solutions at a concentration of 10^−2^ M, then thoroughly washed in UPW and dried in air. Ten spectra were acquired at different positions on the substrate surfaces, with the resulting averaged spectra having been normalized for the exposure time and laser power, and the fluorescence background was subtracted.

## 4. Conclusions

In this paper, we investigated the effect of the wet-chemical functionalization of a dense vertical array of Si nanowires with three alkoxysilanes (APTES, MPTMS, and GPTMS) on the surface binding of Au NPs. For the first time, our experiments show that the most efficient and uniform Si NW decoration is obtained after their surfaces are grafted with GPTMS. Here, we also show that GPTMS-functionalized Si NWs decorated with Au NPs can perform as SERS-active 3D platforms with an enhancement up to a factor of 8 compared to the effects exerted by Au thin films. This industrially compatible and low-cost method for easily fabricating complex SERS-active architectures is very attractive, especially in the frame of their potential integration within Si-based (bio)sensing devices. The homogeneously distributed Au NPs can also provide the Si NWs with new interaction capabilities typical of the decorating noble metal, paving the way to highly sensitive and selective detection of (bio)analytes in hot fields such as biomedical, environmental, and food analysis.

## Figures and Tables

**Table 1:** Average diameter of Au NPs decorating bare and surface-functionalized Si NWs, as obtained from the statistical analysis reported in Figure 4.

Au NPs onto Si NWs. Samples	Average NP Diameter (nm)
Si NW/Au NP	19.2 ± 5.7
APTES-Si NW/Au NP	17.5 ± 6.4
MPTMS-Si NW/Au NP	29.4 ± 11.9
GPTMS-Si NW/Au NP	26.5 ± 8.3

**Table 2:** Amplification of SERS signals for the different Au NP-decorated samples.

Au NPs onto Si NW Samples	I_SERS_/I_Au Film_ @ 450 cm^−1^	I_SERS_/I_Au Film_ @ 1625 cm^−1^	I_SERS_/I_Au Film_ @ 450 cm^−1^	I_SERS_/I_Au Film_ @ 1625 cm^−1^	EF	EF
	λ = 785 nm	λ = 785 nm	λ = 633 nm	λ = 633 nm	λ = 785 nm	λ = 633 nm
GPTMS-Si NW/Au NP	78.7	83.0	63.4	93.0	2.9 @ 450 cm^−1^	10.8 @ 450 cm^−1^
					2.6 @ 1625 cm^−1^	5.3 @ 1625 cm^−1^
Si NW/Au NP	55.9	67.6	39.6	65.3	2.5 @ 450 cm^−1^	10.4 @ 450 cm^−1^
					2.4 @ 1625 cm^−1^	7.4 @ 1625 cm^−1^
GPTMS-Si Bulk/Au NP	31.1	31.6	5.9	17.6		
Si Bulk/Au NP	19.4	27.9	3.8	8.8		

## Data Availability

All data supporting the findings of this study are available from the Corresponding Authors upon request.
